# Positive public attitudes towards agricultural robots

**DOI:** 10.1038/s41598-024-66198-4

**Published:** 2024-07-06

**Authors:** Hendrik Hilmar Zeddies, Gesa Busch, Matin Qaim

**Affiliations:** 1 Center for Development Research (ZEF), University of Bonn, Bonn, Germany; 2 Food Consumption and Wellbeing, Department of Sustainable Agriculture and Energy Systems, University of Applied Sciences Weihenstephan-Triesdorf, Freising, Germany; 3 Institute for Food and Resource Economics, University of Bonn, Bonn, Germany

**Keywords:** Robots, Agriculture, Sustainable food systems, Technology acceptance, Framing, Environmental social sciences, Environmental economics, Psychology and behaviour, Sustainability

## Abstract

Robot technologies could lead to radical changes in farming. But what does the public know and think about agricultural robots? Recent experience with other agricultural technologies—such as plant genetic engineering—shows that public perceptions can influence the pace and direction of innovation, so understanding perceptions and how they are formed is important. Here, we use representative data from an online survey (*n* = 2269) to analyze public attitudes towards crop farming robots in Germany—a country where new farming technologies are sometimes seen with skepticism. While less than half of the survey participants are aware of the use of robots in agriculture, general attitudes are mostly positive and the level of interest is high. A framing experiment suggests that the type of information provided influences attitudes. Information about possible environmental benefits increases positive perceptions more than information about possible food security and labor market effects. These insights can help design communication strategies to promote technology acceptance and sustainable innovation in agriculture.

## Introduction

Intensive farming systems are facing increasing criticism in Europe. Much of the criticism focuses on effects of large-scale farming on the environment and human health^1,2^. Agriculture and food systems are challenged to further increase production to meet the rising global demand for food and biomass and, at the same time, reduce the negative environmental and health impacts as well as food losses and waste^3,4^. In many places—such as Europe and the United States—finding sufficient labor to do the heavy farm work is an additional challenge^5,6^. Robots conducting tasks like precision spraying could help address some of these challenges^5–7^. Robots and other digital technologies promise to make agriculture more precise and sustainable. Sensor-based robots can facilitate more targeted input use, which may help to reduce fertilizer and pesticide quantities as well as soil compaction while enhancing crop yields and biodiversity^8–10^. In an ideal scenario, robot-based farming would increase agricultural output and decrease human labor needs and negative environmental impacts^11^.

So far, robots are not yet widely used in crop farming anywhere in the world. While several applications are being developed and tested, actual adoption rates remain low. Issues of scalability and legal uncertainties, such as strict regulations for autonomous machinery, are currently preventing more widespread dissemination^12,13^. One early example that some farmers have adopted is robots for hoeing and weeding, especially in organic production systems where the use of synthetic herbicides is banned^14^. Farms relying on robots for all field operations without human labor are still a future scenario^11^, even though some projects develop technologies with almost full automation in mind^15^.

With economic development, the share of the labor force employed in agriculture is shrinking. In most high-income countries, very few individuals work in agriculture^16^. This labor force reduction is facilitated by increasing mechanization, with robot technologies possibly representing the next phase^8^. Despite fewer people working in agriculture, public interest in the effects of food production technologies has been growing in many high-income countries, partly driven by food scandals and rising sustainability concerns^17^. New agricultural technologies are often seen with skepticism, as the example of genetically modified (GM) crops demonstrates^17,18^. Even though GM crops can help to make farming more productive and environmentally-friendly, many perceive them as risky and unwanted, especially in Europe^19^. Robots are different because—unlike GM technologies—they do not alter food products directly. However, many people in Europe also care about the social implications of agricultural technologies, and robots may not fully align with common traditional views of farming where humans cultivate the land^8^. Furthermore, crop farming robots tend to collect large amounts of data on the critical infrastructure of food production and could also influence rural employment^8,20^.

Several existing studies analyze public perceptions of robot technologies in agriculture. Early studies looked at milking robots and revealed considerable potential for public disagreement^21,22^. Crop farming robots are different. On the one hand, they do not deal with farm animals, which could increase public acceptance. On the other hand, unlike milking robots, crop robots are not used in closed environments but in open fields, entailing potential interactions with society that cannot be fully controlled by the farmer. A few recent studies examine robots in crop farming, mostly focusing on specific applications^23–25^. These studies suggest that public attitudes are generally quite positive, but that potential for controversy exists^23–25^. Public awareness of robots in crop farming has not been analyzed before. Nor have public perceptions been examined from a broader perspective—beyond specific application examples.

Against this background, better understanding public attitudes towards robot-based farming at an early adoption stage is important since the latency and emergence phases of an issue-attention circle determine how society perceives and debates particular topics^26,27^. Identifying possible concerns early on may also help to improve technological details and public communication, and thus increase society’s awareness and trust and reduce the likelihood of rejection. In this study, we analyze public awareness of robots in crop farming, public attitudes towards such technologies, and how these attitudes are influenced by information and sociodemographic factors.

Our study was conducted in Germany, a country where the public tends to scrutinize farming practices and new technologies like GM crops quite critically^28,29^. We analyze awareness and perceptions of robot-based crop farming with a nationally representative online survey. The robots considered in our research are specified for autonomous operation in agricultural fields, not in road traffic or other public spaces.

## Methods

### Online survey

We developed and implemented an online survey with German residents aged 18 years and older to elicit public attitudes towards agricultural robots. The survey was designed to analyze attitudes from the point of view of citizens, which includes food consumer aspects but also broader sustainability preferences. The survey was conducted between January 5th and 13th, 2023. It was programmed with the software LimeSurvey and hosted on a server of the University of Bonn. The sample was recruited online by a professional panel provider (respondi AG). We informed all participants that their data would be collected anonymously and used solely for scientific research. Participation required active consent of the participants to the data protection agreements of the University of Bonn. The study was reviewed and approved by the Ethics Committee of the Center for Development Research (University of Bonn).

We tested the study design twice before running the actual survey. In November 2022, we interviewed 27 participants using a convenience sample to determine whether the information treatments were well understood. In December 2022, a soft launch was conducted with the panel provider (*n* = 248). The purpose of this soft launch was to test the study’s internal validity and identify any patterns in response behavior that might indicate weaknesses in the study design. Participants in these pre-tests were not included in the final sample for analysis.

### Sample

Quota sampling was used to ensure the sample’s representativeness based on the German adult population. The quota data refer to the long-term market media study program “b4p- Best for planning 2021” in Germany, which has been analyzing media use and consumer behavior since 2013. In total, 2,500 complete questionnaires were collected. On average (median), it took participants 13.5 min to complete the questionnaire. We discarded 231 questionnaires, either because the response time was 50% below the median or the answers displayed no variation in spite of contradictory questions. Therefore, the final sample contains observations from 2,269 participants.

In online surveys, population groups without internet access are automatically excluded, possibly leading to a biased sample. However, in Germany, over 90% of the adult population have internet access, and a large proportion of those without internet access belong to the age category over 60^30^, who are properly represented in our final sample. A small bias may occur because participation was voluntary, and those who signed up may have a greater affinity for technology than the average. However, the quota system tries to ensure a diversified sample in terms of sociodemographic characteristics. We, therefore, claim that our data are nationally representative. Given the sample size, the statistical power is meaningful.

### Survey and experimental design

The survey started with a short general introduction and the consent to participate. Then, before providing any information about agricultural robots, the following items/constructs were surveyed: involvement level with agriculture^31^, trust in farmers, knowledge about crop farming practices, awareness level of robot use in agriculture, and attitude towards agricultural robots.

**Figure 1:**
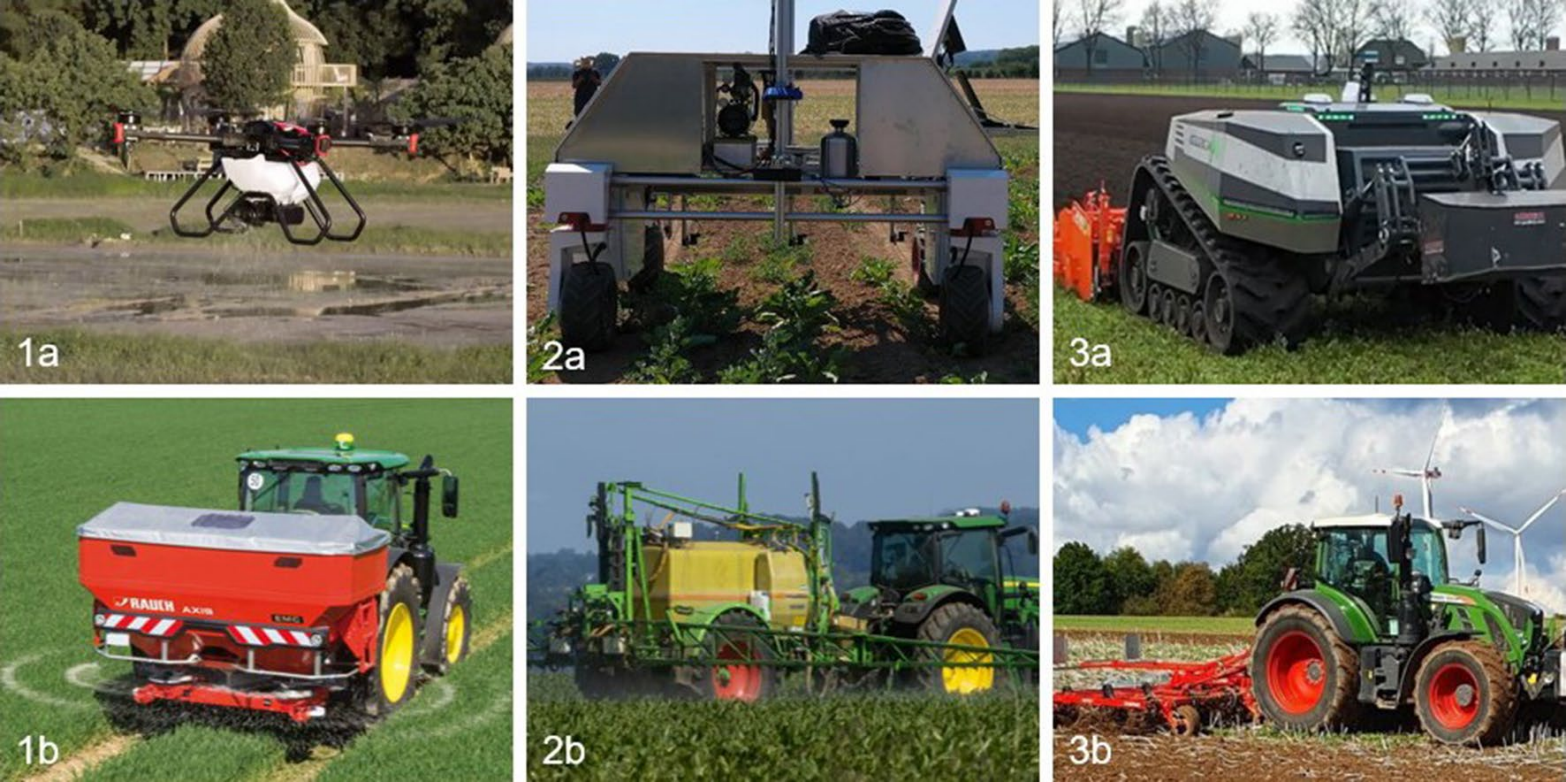
Agricultural robots and conventional alternatives used as examples in the survey and experiment. 1a: Fertilizer-spreading drone (Source: Weber Agrar Robotik GmbH); 1b: Conventional fertilizer-spreading tractor (Source: RAUCH Landmaschinenfabrik GmbH); 2a: Spot-spraying robot (Source: Alireza Ahmadi project PhenoRob); 2b: Conventional field application of pesticides (Source: Mirko Fabian on Unsplash.com); 3a: Autonomous tillage robot (Source: AgXeed BV); 3b: Conventional tractor tilling the field (Source: Authors’ material).

In the second part of the survey, every participant received general information about three agricultural robots and their human-controlled conventional alternatives. The three examples are a drone that applies fertilizer, a spot-spraying robot, and a robot tractor for tillage. The conventional alternatives are a tractor with a mounted fertilizer spreader, an attached sprayer, and a mounted cultivator (Fig. 1).

In order to test the influence of additional information about the likely effects of robots on participants’ attitudes, we developed and implemented a framing experiment as part of the survey. Framing experiments are critically discussed in the literature, as they often produce small effect sizes. A recent meta-analysis suggests that—in spite of small average effect sizes—information effects cannot be ignored in terms of influencing attitudes^32^. In addition, a recent survey-based experiment on climate change and GM food policy preferences underscores the potential effects of information in shaping attitudes^33^.

For our framing experiment, we used a between-subject design and randomly assigned participants to one of three treatment groups or the control group. This resulted in a 4 × 1 study setup. Participants in the three treatment groups received further information about potential positive effects of robots on (1) food security, (2) farm labor facilitation, or (3) environmental outcomes. Participants in the control group received no further information about potential effects of robots. The three treatments are briefly explained below. Further details are provided in Supplementary Information Sect. 1. In the experiment, the information to study participants was provided in German language, both as text and audio files. Here, we provide English translations of the text. Participants were not aware of participating in an experiment with different treatment groups.

The “Food security” treatment emphasizes the positive contribution that robots might offer to secure future food supplies. Greater precision and constant monitoring of crops enable more accurate crop management and, thus, higher and more stable yields. Automation increases cultivation efficiency, reduces losses, and uses operating inputs more effectively^34^. A growing world population that needs to be nourished with limited resources requires efficient food supplies^3^.

The “Labor” treatment emphasizes potential positive effects of robots on agricultural labor markets. Robot technologies facilitate the farm work and reduce physically demanding tasks, thus addressing labor shortages^7,35^ and making farm work more attractive across genders^8^. Demographic trends in high-income countries will likely contribute to rising shortages of agricultural workers in the future^5,6^.

The “Environment” treatment underlines potential environmental benefits of robot technologies. Robots offer the prospect of reducing the environmental impact of agricultural production through more precise and needs-based application of inputs^34^, the protection of the soil^8^ and allowing smaller field sizes and more diversity on the fields and in the farm landscapes^9^.

Using a negative framing could have been interesting as well, but we decided against it because we did not want to spread unsubstantiated negative information about crop farming robots. The limited media coverage of robots used in crop farming is also predominantly positive^36^.

Following the information frames, participants were asked to answer two sets of questions. The first set contained general assessment statements on the potential benefits of robot technologies. The second set assessed participants’ attitudes, which we explain in more detail below. The questionnaire continued with the last information part, in which we informed every participant about agricultural robots’ potential risks concerning data sovereignty, which we deemed necessary for participants unfamiliar with data collection patterns of agricultural machinery^20^. Afterwards, participants determined their level of concern for different potential risks of agricultural robots.

Questions about attitudes and concerns were asked by providing statements that study participants could agree or disagree with. We used five-point Likert scales coded as follows: 1- “Strongly disagree”, 2- “Rather disagree”, 3- “Partly/Partly”, 4- “Rather agree”, 5- “Strongly agree”. Beyond attitudes about robots, we also asked participants about various other types of preferences, habits, and sociodemographic characteristics. Preference for organic farming measured as shopping frequency was assessed on a five-point Likert scale, ranging from 1—“Never” to 5—“Always”. Proximity to farming is defined as high if participants have friends, relatives, neighbors, or other personal contacts employed in agriculture. Proximity to farming is defined as low if participants have contact with agriculture through purchases in a farm shop or holidays on a farm. No contact means that participants have no personal connection to farmers. These and other variables are used in the analysis to explain attitudes (see below).

### Knowledge about farming

We were interested in participants’ knowledge about farming as this can influence perceptions of agricultural technologies. Subjective knowledge (self-assessed) and objective knowledge (actual) can differ considerably^37^. For this reason, we surveyed knowledge using a quiz. Study participants were confronted with eight statements regarding crop production in Germany. Four of these statements were true; the others were false (Supplementary Information Table S1). Participants had to select whether they thought the statement was true or false. Based on the responses, we created three knowledge categories: “low” for 0–2 correct answers, “medium” for 3–5 correct answers, and “high” for 6–8 correct answers. At the end of the survey, we provided study participants with the quiz solution. In addition to capturing objective knowledge, we also asked respondents to self-assess their knowledge.

### Attitude constructs

**Figure 2:**
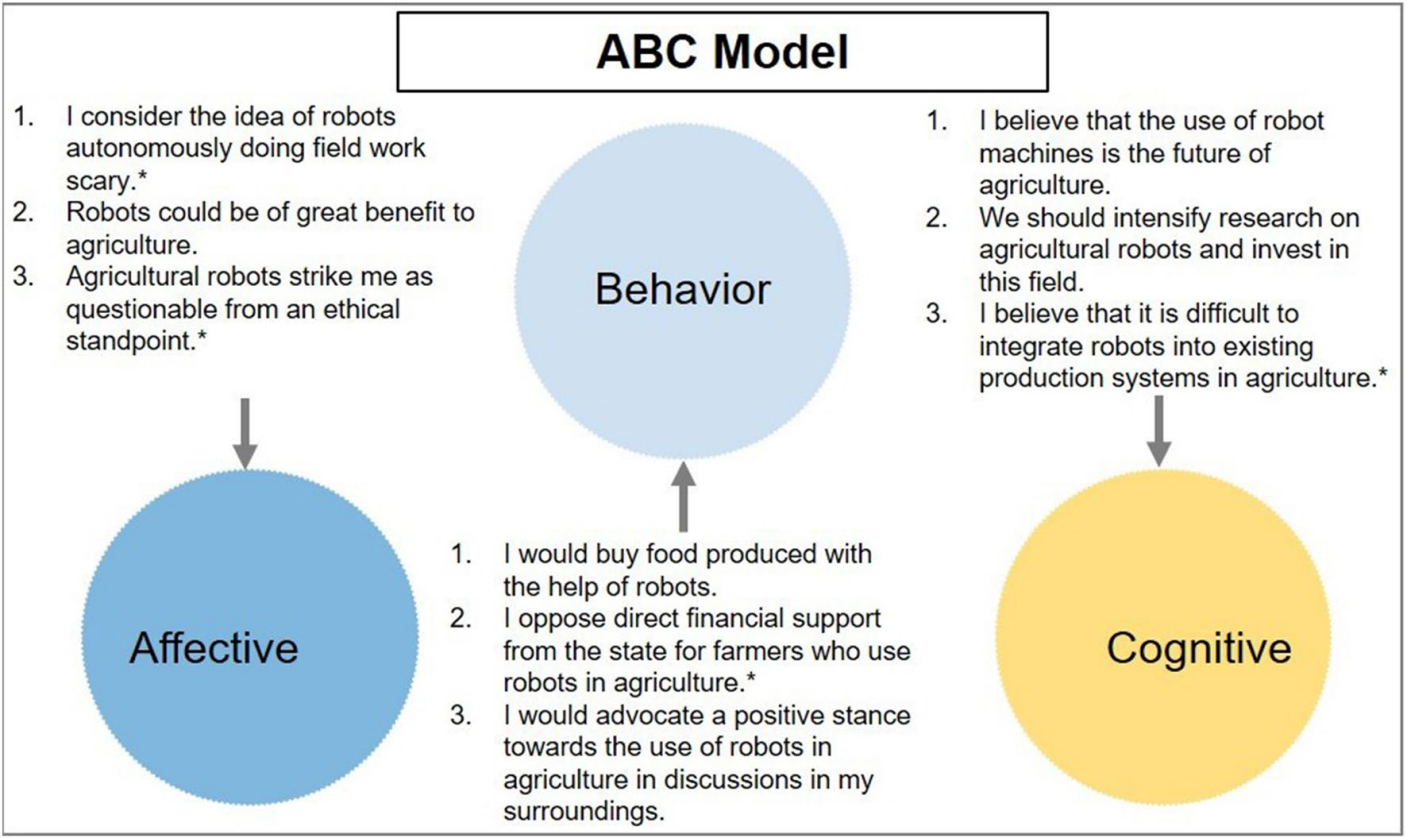
Composition of the ABC model (*Original statement with negative polarization and re-coded for the model).

We use two variables to assess respondents’ attitudes towards agricultural robots. The first variable is based on the “ABC model of attitude” derived from psychology^38,39^. The ABC model is widely used in marketing studies^40^. According to this model, the attitude of an individual depends on an affective (A), a behavioral (B), and a cognitive component (C). We developed a set of statements for each component and used principal component analysis (PCA, see below) to condense the information obtained into one attitudinal construct named “ABC”. No validated scale exists to evaluate the ABC components for agricultural robots. We base our model design on Mathew^41^ and interpret behavior (B) as indirect interaction with agricultural robots. The concrete design of the statements builds on the existing literature in other areas^28,42,43^. Figure 2 presents a detailed overview of the statements used.

The second attitude variable is based on the direct question: “Should robots like the ones you have just seen be used in agriculture?” We refer to this variable as “Attitude”. By using two different approaches/variables to measure attitudes, we cross-check the results and increase their reliability.

### Statistical analysis

The statistical analyses were performed with the software STATA (Version 16.1). In addition, we used SPSS (Version 28) for the PCA to construct the “ABC” variable.

#### Descriptive analysis

We analyze respondents’ awareness of agricultural robots and their general attitudes towards farming technologies using descriptive statistics for the whole sample and for various sociodemographic groups. We also examine whether the responses vary significantly between the different groups, using the non-parametric Kruskal–Wallis test, which offers more freedom with respect to distribution assumptions than alternative parametric tests^44^. We also compare the groups pairwise using Dunn’s test. The results are Bonferroni corrected. For some of the variables, we reduced data complexity by extracting constructs according to the theoretical constructs based on the literature. This applies to the constructs of agricultural involvement, the ABC model, and climate-conscious food purchasing behavior^31,38,45^.

#### Construct validity

For the ABC model, we performed a PCA using the Varimax rotation. As the ABC model had not been applied previously to analyze attitudes towards agricultural robots, we could not determine a priori whether the extracted questions fit together to form one construct or whether an exploratory analysis on the three subdomains would produce three constructs (affective, behavioral, and cognitive). The PCA analysis resulted in a highly significant single construct for the ABC model (Supplementary Information Tables S2a and S2b). Cut-offs were set at > 0.500 for the factor loadings and > 0.600 for the Kaiser–Meyer–Olkin quality criterion.

All constructs underwent reliability and internal consistency checks. The cut-off criteria were set as follows: average variance extracted > 50%, Cronbach’s *α* > 0.600, and McDonald’s *ω* > 0.600. The respective construct variables were saved as unweighted average scores to align the scaling with the underlying variables and to simplify interpretation^46^. Since the “ABC” variable is one of the dependent variables in the regression analysis (see below), we applied different calculation methods. In addition to the unweighted average, in a further step, the ABC variable was re-transformed to the original scale metric after calculating the average value by rounding the values (rounded factor scores). We also calculated the Anderson-Rubin score of the ABC factor, an orthogonal solution resulting in unbiased coefficients^46^.

#### Regression models

We use regression models to analyze how the information treatments and various sociodemographic factors influence attitudes towards agricultural robots. Due to the ordinal structure of our dependent variables (“ABC” and “Attitude”), we use an ordinal logistic regression estimator. The estimated odds ratios can be interpreted as the probability that participants select a one-category higher level of the underlying scale^47^. In addition, we use the estimation results to calculate marginal effects. A Brant test that we conducted led to insignificant results. Hence, we conclude that the parallel lines assumption holds^47^.

A general equation of our regression models is:

1 $${Y}_{i}^{*}= {\beta }_{0}+ {\beta }_{1}{Fs}_{i}+ {\beta }_{2}{Lab}_{i}+{\beta }_{3}{Env}_{i}+ {\beta }_{x}{X}_{ij}+ {\varepsilon }_{i},$$

where ${Y}_{i}^{*}$ is attitude of individual $i$ towards robots, measured either with the ABC construct or with the direct attitude question. The * indicates that the dependent variables are measured on a discrete scale with $k=5$ categories. ${\varepsilon }_{i}$ is a random error term, and ${\beta }_{0}$ to ${\beta }_{x}$ are coefficients to be estimated. We are particularly interested in the effects of the information treatments, expressed as dummies, with ${Fs}_{i}$ denoting the food security treatment, ${Lab}_{i}$ the labor treatment, and ${Env}_{i}$ the environment treatment (the control group is the baseline). ${X}_{ij}$ is a vector of sociodemographic control variables, including age, gender, education, income, location, trust in farmers, involvement with agriculture, climate-conscious food purchasing behavior, organic preference, knowledge about farming, and proximity to farmers.

The ordinal logistic estimator has several advantages over OLS for our data and models. OLS potentially underestimates the influence of the explanatory variables due to the ordinal structure of the outcomes. Furthermore, the OLS estimates cannot be used for effect size interpretations^48^. Nevertheless, we use an OLS estimator as a robustness check of the direction of the estimates and their levels of statistical significance.

## Results

Table 1 shows sociodemographic statistics for our total sample of 2,269 respondents in comparison to the German average, confirming that our sample can be considered nationally representative (the distribution of the sample by German states is shown in Supplementary Information Table S3). Table 1 also compares the three experimental treatment groups and the control. We find no significant differences between these groups, which is not surprising given that the treatments were allocated randomly and we used quota sampling.

**Table 1:** Sample description.

	By experimental treatment group				Total sampleoverall	Germany (national statistic)
	Control	Food security	Labor	Environment		
Age						
18–30	101 (17.7)	107 (18.7)	105 (18.6)	100 (17.8)	413 (18.2)	18%
31–40	92 (16.1)	95 (16.6)	80 (14.2)	89 (15.9)	356 (15.7)	17%
41–50	88 (15.4)	88 (15.4)	89 (15.8)	86 (15.3)	351 (15.5)	16%
51–60	134 (23.5)	122 (21.3)	137 (24.3)	126 (22.5)	519 (22.9)	21%
61–93	156 (27.3)	161 (28.1)	153 (27.1)	160 (28.5)	630 (27.8)	28%
χ^2^-Test	Pearson χ^2^ = 2.9912 *P* = 0.996				–	–
Sex						
Male	283 (49.6)	282 (49.2)	277 (49.1)	276 (49.2)	1,118 (49.4)	50%
Female	288 (50.4)	291 (50.8)	285 (50.5)	282 (50.3)	1,146 (50.6)	50%
Divers	0	0	2 (0.4)	3 (0.5)	5 (0.2)	n.a
χ^2^-Test	Pearson χ^2^ = 5.5281 *P* = 0.478				–	–
Education						
No qualification/SNVQ^1^	185 (32.4)	185 (32.3)	182 (32.3)	177 (31.6)	729 (32.3)	31%
Secondary school VQ^2^	177 (31.0)	186 (32.5)	181 (32.1)	180 (32.1)	724 (32.1)	32%
High school (Abitur)^3^	205 (35.9)	200 (34.9)	199 (35.3)	199 (35.5)	803 (35.6)	36%
χ^2^-Test	Pearson χ^2^ = 8.0632 *P* = 0.528				–	–
Income						
< 1,500 €	91 (15.9)	90 (15.7)	93 (16.5)	92 (16.4)	366 (16.1)	15%
1,501–3,000 €	192 (33.6)	194 (33.9)	186 (33.0)	185 (33.0)	757 (33.4)	34%
3,001–4,500 €	173 (30.3)	174 (30.4)	164 (29.1)	168 (29.9)	679 (29.9)	30%
> 4,501 €	115 (20.1)	115 (20.1)	121 (21.5)	116 (20.7)	467 (20.6)	20%
χ^2^-Test	Pearson χ^2^ = .7880 *P* = 1.000				–	–
*n*	571	573	564	561	2,269	–

Pearson χ^2^ test of independence: Significant differences occur at a Probability (*P*) level < 0.05.

Total numbers of respondents, share of respondents per group, and category in parenthesis.

^1^SNVQ = Secondary school non-vocational qualification, Corresponds to the German “Hauptschulabschluss” (low education); ^2^VQ = Vocational qualification, Corresponds to the German “Realschulabschluss”; ^3^High education.

### Awareness and attitudes before the information treatments

**Figure 3:**
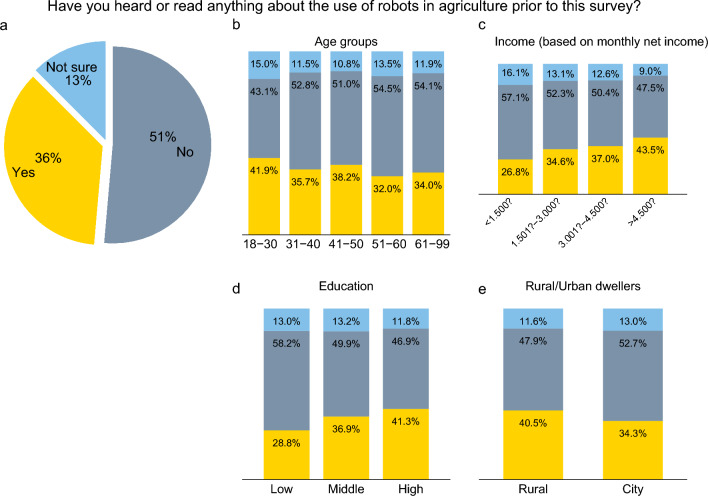
Awareness of agricultural robots. The question was asked before any information about agricultural robots was provided (*n* = 2,269). Tests for statistical significance between the different groups are shown in Supplementary Information Table S4.

Before providing any information about agricultural robots, study participants were asked about their awareness and general attitudes towards agriculture. Figure 3 shows responses to the question whether or not the individual had heard or read anything about the use of robots in agriculture before participating in this study. Figure 3a reveals the overall response behavior: 51% of the participants had not heard or read anything about the use of robots in agriculture before the survey, whereas 13% were unsure. Only 36% mentioned that they had heard or read anything about agricultural robots before.

Figures 3b–e show responses to the same question, but now differentiating by sociodemographic groups (differences in the responses between groups are tested for statistical significance in Supplementary Information Table S4). As can be seen, awareness of robots is unevenly distributed across the population. Young individuals below the age of 30 are significantly more likely to have heard about agricultural robots than older individuals (Fig. 3b). For the other age groups, no significant differences are observed. Education and income are also significantly related to awareness. Awareness levels are considerably higher among the richer and better-educated individuals than among the poorer and less-educated ones (Fig. 3c,d). Furthermore, individuals living in rural areas are more aware of agricultural robots than individuals living in urban areas (Fig. 3e).

**Figure 4:**
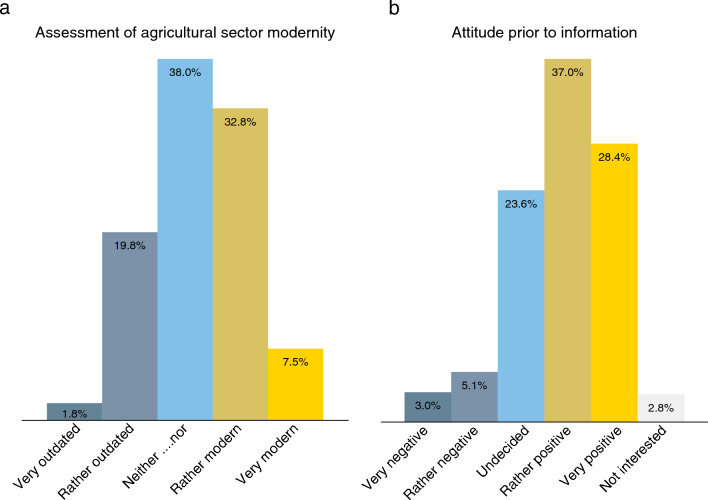
Perceptions of agriculture and attitudes towards agricultural robots. (**a**) Perception of modernity of the agricultural sector, based on the question: I perceive agriculture with regard to digitalization and technical innovations as…. (*n* = 2,269). (**b**) Attitudes towards agricultural robots, based on the question: How do you feel about robots being used in agriculture? (*n* = 2,269). Both questions were asked prior to providing any information about agricultural robots.

After determining the level of awareness, we asked participants to assess the modernity of the agricultural sector and their general attitude towards the use of robots in agriculture. These questions were also asked before providing any information about robot technologies. Around 40% of the participants consider agriculture a rather modern or very modern sector, whereas 22% consider the sector rather outdated or very outdated (Fig. 4a). These are affective assessments, as we did not specify a concrete other sector for comparison.

Individual’s interest in agricultural robots is high: less than 3% state that they are not interested in this topic (Fig. 4b). Being interested does not necessarily mean that attitudes are positive, but 65% state that they feel very positive or rather positive about the use of robots in agriculture. Only 8% express negative feelings. Close to one-quarter of the participants are uncertain whether they feel positive or negative about robots being used in agriculture.

### Effects of information treatments

**Figure 5:**
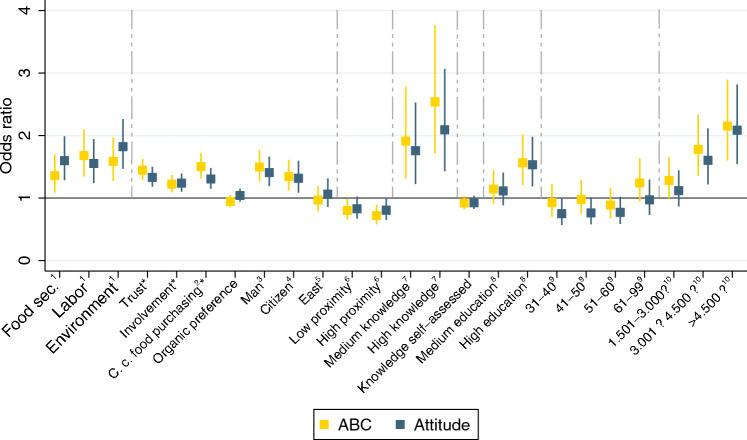
Effects of information treatments and sociodemographic factors on attitudes towards agricultural robots. Results based on ordered logistic regression models with attitude as dependent variable measured in terms of a five-point Likert scale, ranging from 1: “Strongly disagree” to 5: “Strongly agree”. Effects are shown as odds ratios with 95% confidence intervals (*n* = 2,251). Two different models are estimated, one with “ABC” (ABC-Model factor score) and one with “Attitude” (direct response to attitude question) as dependent variable. Further details of the regressions are shown in Supplementary Information Table S5. ^1^Base category = Control group; ^2^C.c. food purchasing = Climate-conscious food purchasing behavior; ^3^Base category = Women; ^4^Base category = Rural Dwellers; ^5^Participants living in East Germany; Base category = West German residents; ^6^Base category = No contact (Low = Contact via farm shops and farm holidays; High = Contact via job, family or friends); ^7^Base category = Low knowledge (based on quiz results: 0–2 = low, 3–5 = middle 6–8 = high); ^8^Base category = No school-leaving qualification/secondary school; ^9^Age: Base category = 18–30; ^10^Income: Base category =  < 1.500€; *Used as average factor score.

Figure 5 presents the regression results. As explained above, the regression models analyze the effects of the information treatments and of relevant sociodemographic characteristics on attitudes towards agricultural robots. One regression uses the ABC model as dependent variable, while the other uses the direct responses to the attitude question. Both dependent variables were surveyed after the information treatments.

We start by looking at the effects of the information treatments, which are shown as the first three variables in Fig. 5. All three treatments contribute significantly to more positive attitudes towards agricultural robots relative to the control group. Among the three treatments, the information about environmental benefits has the largest positive effect on attitudes. The odds ratios shown in Fig. 5 indicate that participants receiving the environmental information treatment are 1.59 (ABC model) and 1.82 (Attitude model) times more likely to rate agricultural robots more positively by at least one unit on the Likert scale than participants in the control group. For the other two treatments, the effects are also positive and significant but somewhat smaller in magnitude.

In addition to the odds ratios illustrated in Fig. 5, Table 2 presents the marginal effects of the information treatments for each attitude response category. As can be seen, the likelihood of observing negative and neutral attitudes towards agricultural robots (lower response categories) is significantly reduced through all three information treatments, whereas the likelihood of observing category five (“Strongly agree”) is significantly increased. In particular, the environmental treatment increases the likelihood of participants choosing category five by 8 percentage points in the ABC model and by almost 13 percentage points in the attitude model. The full distributions of all attitude responses in the treatment and control groups are shown in Supplementary Information Table S6.

**Table 2:** Marginal effects of the information treatments.

Variable	ME Category 1	ME Category 2	ME Category 3	ME Category 4	ME Category 5
ABC					
Food security	− 0.005*	− 0.012**	− 0.040**	0.006	0.051***
Labor	− 0.007***	− 0.020***	− 0.066**	0.002	0.091***
Environmental	− 0.007***	− 0.018***	− 0.059***	0.003	0.080***
Attitude					
Food security	− 0.008***	− 0.016***	− 0.056***	− 0.018**	0.098***
Labor	− 0.007**	− 0.015***	− 0.053***	− 0.016**	0.091***
Environmental	− 0.009***	− 0.020***	− 0.070***	− 0.028***	0.127***

Marginal effects (ME) of the treatments in comparison to the control group are shown. ME calculated based on ordered logistic regressions with attitude as dependent variable measured in terms of a five-point Likert scale, ranging from category 1: “Strongly disagree” to 5: “Strongly agree”. Two different models were estimated, one with the ABC model factor score and one with a direct response to the attitude question as dependent variable. *, **, and *** indicate significance at the 5%, 1%, and 0.1% level, respectively.

### Effects of sociodemographic factors on attitudes

The influence of the sociodemographic control variables on attitudes towards agricultural robots, as estimated from the regression models, is also shown in Fig. 5. Many of these sociodemographic factors are also significantly associated with attitudes. Higher levels of education and income are positively associated with attitudes, as are trust in farmers, involvement (interest) in agriculture, urban location, and being male. The age of individuals does not influence attitudes towards agricultural robots. Somewhat surprisingly, individuals with high personal proximity to farming (e.g., having relatives or friends who are farmers) are more skeptical towards agricultural robots than individuals without personal contact to farmers. This may be related to concerns that robots could profoundly change the lives of farmers, which is more relevant for individuals with high proximity to farming.

Organic food preferences do not seem to influence individual’s acceptance of agricultural robots, whereas individuals who claim to purchase food in a climate-conscious way have above-average acceptance levels. Also, we observe a significantly positive relationship between objective knowledge about farming and attitudes towards robots, whereas for self-assessed knowledge we find no significant relationship.

Marginal effects of these sociodemographic factors are presented in Supplementary Information Table S7. Estimation results with OLS instead of ordered logistic models are shown in Supplementary Information Table S5. These alternative estimates confirm the same directions and levels of statistical significance, thus underscoring the robustness of the findings.

### Evaluation of potential issues regarding robots

**Figure 6:**
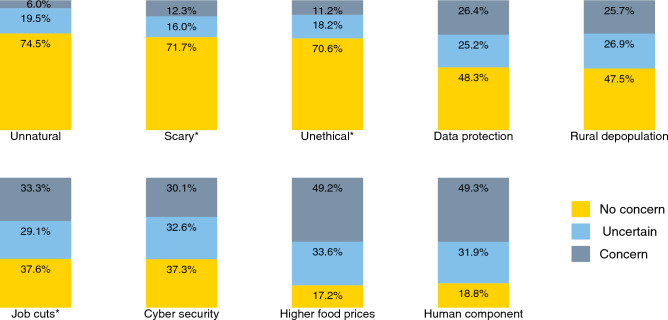
Possible concerns about agricultural robots. All areas of possible concern are measured on a five-point Likert scale (*n* = 2,269). For the graphs, we grouped 1 (“Strongly disagree”) and 2 (“Rather disagree”) into the category “No concern”, and 4 (“Rather agree”) and 5 (“Strongly agree”) into the category “Concern”. * indicates a significant Kruskal–Wallis test and a significant difference between at least one treatment group and the control group, as shown in Supplementary Information Table S8.

Beyond the general question of whether or not robots should be used in crop farming, there may be specific areas of concern worth exploring. Study participants were asked to rate specific areas of possible concern, as shown in Fig. 6. Since these ratings were done after the information treatments, we also tested for possible differences between the treatment and control groups (Supplementary Information Table S8). The observed differences are small, so the results in Fig. 6 refer to the full sample.

For several of the areas shown in Fig. 6, levels of concern seem to be relatively low. Only 6% consider the use of robots in farming or the foods produced with robots as unnatural, whereas 74.5% disagree, meaning that robots seem compatible with “naturalness” for the large majority. Similarly, more than 70% of the participants disagree with the statement that agricultural robots are scary or unethical to use.

Concerns about data protection are somewhat more widespread. Less than 50% of the respondents are not concerned about data protection issues, whereas the rest are either concerned or at least uncertain. In this context, we should note that we informed all participants that questions around data ownership between farmers and machine manufacturers still need to be clarified and regulated^20^. Related to data protection is the potential issue of cyber security, where over 60% are either concerned or uncertain. Only 37% stated that they are not concerned about cyber security risks. In the survey, we contextualized cyber security issues as the increased degree of interconnectedness of machines and the associated risk of cybercrime, such as hacking attacks.

Figure 6 also reveals some concerns related to job losses and rural depopulation through agricultural robots. However, the biggest concerns are observed in terms of possible implications of robots for food prices and lack of human involvement in food production. Half of the study participants share the concern that the use of robots may lead to food price increases due to high costs of the technology, with another third expressing uncertainty. Likewise, half of the participants are concerned about a missing human component in food production.

## Discussion

Our study provides insights into public awareness of robots in agriculture, as well as societal expectations and concerns and how perceptions are shaped. These insights may help to improve science communication for sustainable innovation. Results reveal a predominantly positive attitude towards robot use in agriculture among German residents. These positive attitudes are in line with a few previous studies that looked at specific robot applications^23–25^.

However, general awareness levels have not been analyzed previously. Our results reveal that awareness of agricultural robots is quite low in Germany, even though other agricultural technologies and issues of farming practices are critically debated by the public^28,29,49^. Over 50% of our survey respondents had never heard about agricultural robots before participating in this study. Individuals with low education levels are less likely to be aware of robots than the average. Even though one might expect individuals with low education levels also to be less interested in this topic, this is not what we observe. In fact, we find high levels of interest in agricultural robots across all population groups. Hence, we conclude that the low awareness levels are rather a reflection of low coverage of the topic of agricultural robots in mainstream media. Communication could be intensified and improved, which may be an important precondition for public acceptance. Farmers and the agricultural industry in Europe often complain about society's prejudices against modern technology and consumers' alienation from the reality of agricultural production, resulting in a notion that constructive communication makes little sense^50,51^. We find no indication of general public prejudices against agricultural robots.

Results from our framing experiment show that additional information significantly affects individual attitudes towards agricultural robots. In our study, the information provided was positive. In particular, building on the literature about the effects of agricultural robots, we provided information on possible benefits in terms of food security, reduced labor needs, and reduced environmental impacts of farming^3,8,9,34,35^. This information further increased technology approval rates. In the experiment, we did not sketch a scenario with explicitly negative effects. However, given low public awareness levels, large interest in the topic, and the fact that additional information affects attitudes, it is likely that negative information could also lead to low acceptance rates of agricultural robots. In other words, there is a certain risk of negative narratives spoiling generally positive attitudes, which might be exploited by certain interest groups trying to prevent further digitalization and automation in agriculture. This makes science-based communication at this early stage of the technology even more important.

Comparing the effects of the different information treatments, we found the largest positive effects on attitudes towards robots for the “environment” treatment, in which possible environmental benefits were emphasized. This result does not surprise because German society is particularly concerned about the negative environmental impacts of farming^23^. Robots may not only contribute to a reduction in fertilizer and pesticide use but may also reduce soil compaction and allow smaller field sizes to be managed efficiently, which can enhance landscape-level biodiversity^8,9,34^. Public outreach activities by science, industry, and media related to robot use in agriculture already stress the potential environmental benefits^36^, which seems to be the right strategy to promote public technology acceptance.

In spite of somewhat smaller effect sizes, the other two information treatments—i.e., “food security” and “labor”—lead to positive effects on individual attitudes towards robots as well. The relatively high technology approval rates in the labor treatment may be surprising at first sight because increased automation in agriculture could imply a loss of jobs^35^. Yet, in recent years, many sectors in Germany, including the farming sector, have been struggling to find sufficient labor^52^, a fact that may be reflected in the responses of study participants. Against the background of labor shortages and expected demographic developments, new automation technologies are seen as an economic opportunity rather than a threat to jobs^53^. While this was shown in other sectors^53^, it was not previously analyzed for the agricultural sector. Our findings clearly suggest that individuals in Germany do not broadly reject the partial replacement of human labor by robots in food production. However, it should be stressed that in Germany, the agricultural sector is highly mechanized anyway^54^ and that only a very small fraction of the population actually works in this sector^55^. In countries with a larger share of the workforce still active in agriculture—such as in large parts of Africa and Asia^16^—concerns about the effects of agricultural robots on employment may be different.

In addition to information, various sociodemographic characteristics—including values and beliefs—also influence individual attitudes towards technology in agriculture^56^. Our results show that trust in farmers, interest in agriculture, knowledge of farming conditions, and general education levels are all associated with more positive attitudes towards agricultural robots. These findings align with previous studies investigating acceptance factors for other new food technologies^17,23,57^.

Understanding potential societal concerns about robot use in agriculture was one of the main motivations for this study. Overall, public levels of concern seem to be limited. While previous studies found that ethical concerns with respect to autonomous technologies matter in the livestock sector^21,58^, our study results indicate that the large majority of participants are neither afraid of robots in crop farming nor do they consider them ethically critical or judge the foods produced by robots as unnatural. The often highly critical views of German citizens and consumers on new food technologies are well documented^28,29,49^. Therefore, we argue that our results from Germany on generally positive perceptions of agricultural robots may also apply to other high-income countries.

Areas of public concern that seem to be somewhat more relevant with respect to agricultural robots are possible issues around data protection, cyber security, food prices, and lacking human involvement in food production. Issues of data and cyber security are not only relevant for farmers but also for society, as cyber-attacks on agricultural robots could potentially affect food security and food safety^20^. Furthermore, field robots collect potentially sensitive data on infrastructure and food production. Data security related to agricultural robots is discussed intensively among experts and will need to be properly regulated^8,20^. Potential issues of cyber security need policy attention, such that data transmission and agricultural production can continue even when critical infrastructure breaks down^59^. Appropriate solutions are important in order to gain public trust and acceptance of robot technologies.

Regarding food prices, half of the study participants are concerned that expensive robot technologies will result in higher food prices. This effect is rather unlikely. Farmers will adopt robot technologies only if they are cost-effective. So far, robots are still too expensive for most farmers, which is one reason for the low adoption rates^13^. Wider adoption can only be expected when the costs of robots decrease or when the cost of human labor increases dramatically. Robots, as such, will hardly lead to rising food prices, which should be clearly communicated to dispel public concerns.

However, farmers' willingness to adopt robots cannot be attributed to economic factors alone. Perceived social norms, including perceptions of control, are also potentially important factors influencing adoption^12^. Perceived control by farmers also appears to be an important technology acceptance factor for consumers and citizens, as our study results suggest. Study participants seem to have no problem with a partial replacement of human labor by robots in food production but seem to be more concerned about a complete replacement. Some minimum human involvement and control may be reassuring in order not to be fully dependent on robots. It should be noted that a food production scenario without any human involvement is not to be expected in the foreseeable future^8^.

A few limitations of our study approach should be discussed. First, attitudes cannot only be influenced by written or oral information but also by the images used in the survey questionnaire (e.g., the size of machines shown). For this reason, we selected different examples to cover a range of possible robot applications in farmers’ fields. The images and explanations were kept as neutral as possible. Nevertheless, it is possible that a different presentation would lead to somewhat different results. Second, participants in an online study cannot evaluate the technologies described under actual production conditions. Third, while we looked at the effects of different types of information on average attitudes towards robots, we did not analyze whether the information treatments have heterogeneous effects on specific population groups (e.g., differentiating by education, income, rural/urban, etc.). Understanding effect heterogeneity may be useful for tailoring and targeting communication campaigns. Fourth, we decided not to use any negative framing, meaning that our statements on possible effects of negative information remain untested. Finally, while some of our general findings may also hold for other high-income countries, attitudes depend on socioeconomic and cultural contexts, meaning that our specific results cannot be generalized. Follow-up research is needed to better understand the links between agricultural robots, farmers’ adoption, policy regulations, environmental and socioeconomic effects, and public acceptance under various conditions.

In conclusion, public perceptions of agricultural robots are generally positive in a German context, which is in line with previous findings^23–25^. In society’s views, robots used in crop farming seem to be compatible with preferences for more environmentally-friendly agriculture, naturalness, and perceived social norms—all aspects that are known to be guiding principles for the acceptance of agricultural practices and innovations^12,25,60^. Hence, there is no reason to fear widespread public rejection of robot technologies in crop farming. However, levels of public awareness and knowledge about agricultural robots are still low, and a few specific concerns exist. We have shown that additional information can influence attitudes significantly. Therefore, it is important to avoid the spread of narratives that primarily focus on risks and concerns, as has happened for GM crops and a few other agricultural practices, resulting in low public acceptance^19,28,29^. Areas of concern need to be addressed, but at the same time, the large potentials of agricultural robots also need to be explained. Objective and balanced science communication is important to promote sustainable innovation in agriculture.

## Supplementary Information

Supplementary Information.

## Data Availability

The data underlying this article are available at the PhenoRob database “Phenoroam”: https://phenoroam.phenorob.de/geonetwork/srv/eng/catalog.search#/metadata/aa1452d8-af03-4513-8cbe-38aca0a6081f
