# Supplementary material for: Positive public attitudes towards agricultural robots
Source: Sci Rep. 2024 Jul 6;14:15607. doi: 10.1038/s41598-024-66198-4 (PMC11227594; doi:10.1038/s41598-024-66198-4)
Supplement: Supplementary file 1 — Supplementary Information. [file 41598_2024_66198_MOESM1_ESM.pdf]

Positive public attitudes towards agricultural robots

Supplementary Information

Hendrik Hilmar Zeddies*, Gesa Busch, Matin Qaim

*Corresponding Author (hzeddies@uni-bonn.de)

Section 1: Information treatments

General information treatment

The English text is a translation of the German original (shown below the translation), which was used in the online study. The picture references refer to Figure 1 of the main article to ensure comprehensibility.

You will now receive explanations, images, and examples of the use of robots in agriculture. You can read the texts or listen to them as audio. Please read the respective text carefully or listen to the attached audio file carefully. If you decide to listen to the audio file, please scroll through the text while listening, as the respective pictures can be found there.

German: Sie erhalten nun Erklärungen, Bildmaterial und Beispiele für den Einsatz von Robotern in der Landwirtschaft. Zum Vergleich zeigen wir Ihnen die aktuell eingesetzte Technik ohne den Einsatz von Robotern. Sie können die Texte dazu lesen oder sich diese auch als Audio anhören. Bitte lesen Sie den jeweiligen Text aufmerksam bzw. hören der beigefügten Audiodatei aufmerksam zu.
Sollten Sie sich für die Audiodatei entscheiden, scrollen Sie bitte beim Zuhören durch den Text, da dort die jeweiligen Bilder zu finden sind.

In the following, you will see examples of agricultural robots that can be used in arable farming. In other words, in crops that are cultivated on a large scale in Germany (e.g., wheat, corn, rapeseed, and sugar beets). The machines perform different tasks.

German: Im Folgenden sehen Sie Beispiele für landwirtschaftliche Roboter, die im Ackerbau eingesetzt werden können. Das heißt, in Kulturen, die in Deutschland großflächig angebaut werden (z.B. Weizen, Mais, Raps und Zuckerrüben). Die Maschinen führen unterschiedliche Aufgaben aus.

(Note: Picture used are shown in Figure 1 of the main paper)

Picture 1a shows a drone fertilizing a field.

German: Bild 1a zeigt eine Drohne, die ein Feld düngt.

For comparison, see how this is done conventionally, with a fertilizer spreader attached to a tractor (Picture 1b).

German: Zum Vergleich sehen Sie, wie dies bisher gemacht wird, mit einem Düngerstreuer, der an einen Traktor angehängt ist (Bild 1b).

Picture 2a shows a robot that uses a camera to detect weeds. The robot then sprays only the identified weeds with herbicides for weed control.

German: Bild 2a zeigt einen Roboter, der mithilfe einer Kamera Unkräuter erkennt. Der Roboter besprüht dann nur die identifizierten Unkräuter mit Herbiziden zur Unkrautbekämpfung.

The usual technique for this is the crop protection sprayer pulled by a tractor (Picture 2b). Here, the entire area is sprayed with herbicides.

German: Die übliche Technik hierfür ist die Pflanzenschutzspritze, die von einem Trecker gezogen wird, wobei hier die gesamte Fläche mit Herbiziden behandelt wird (Bild 2b).

Picture 3a shows a robotic tractor driving autonomously and tilling the soil.

Bild 3a zeigt einen allein fahrenden Traktorroboter bei der Bodenbearbeitung.

The usual counterpart is larger and requires a driver (Picture 3b).

German: Das übliche Gegenstück ist größer und benötigt einen Fahrer (Bild 3b).

All robot examples are characterized by the fact that the systems can perform tasks independently in the field based on a defined algorithm independently of human control after respective programming by the farmer**.**

German: Alle Roboterbeispiele zeichnen sich dadurch aus, dass die Systeme die Aufgaben anhand eines definierten Algorithmus eigenständig, ohne menschliche Steuerung, auf dem Feld ausführen können.

Information treatment 1: Food security

Agricultural robots offer great potential to enhance the efficiency of agricultural crop production and provide a secure food supply for the world’s growing population. Robots can detect diseases and missing nutrients faster. This reduces losses due to fungi, other pests, or nutrient deficiencies. Crop yields could be increased as a result. Against the background of a growing world population that needs to be nourished, such a reduction in crop losses is becoming increasingly important.

German: Landwirtschaftliche Roboter besitzen großes Potenzial, die landwirtschaftliche Pflanzenproduktion effizienter zu gestalten und die steigende Weltbevölkerung besser zu versorgen. Roboter können Krankheiten und fehlende Nährstoffe schneller erkennen. Dadurch sinken Verluste durch

Pilze, andere Schädlinge oder Nährstoffdefizite. Die Erntemengen könnten hierdurch gesteigert werden. Vor dem Hintergrund einer steigenden Weltbevölkerung, die ernährt werden muss, wird eine solche Reduktion von Ernteverlusten immer wichtiger.

Information treatment 2: Labor

Agricultural robots have great potential to make work in the field easier and more attractive. Many robots perform tasks in the fields on their own and only need to be programmed by the farmer. If robots are used more and more in agriculture, this will reduce the need for human labor time to grow crops. This could be an advantage for agriculture because it is currently difficult to find enough workers on the farms. Fewer and fewer people are willing to perform physical labor and long workdays in mostly sparsely populated regions of Germany. Robots offer the possibility to replace this missing workforce. The possible physical relief for farmers through robots means that the industry could become more attractive across genders.

German: Landwirtschaftliche Roboter besitzen großes Potenzial, Arbeiten auf dem Feld zu erleichtern und attraktiver zu machen. Viele Roboter führen die Aufgaben auf den Feldern alleine durch und müssen nur noch vom Landwirt/ der Landwirtin programmiert werden. Wenn Roboter in der Landwirtschaft mehr und mehr eingesetzt werden, sinkt dadurch der Bedarf an menschlicher Arbeitszeit für den Anbau von Lebensmitteln. Für die Landwirtschaft wäre dies ein Vorteil, denn es ist aktuell schwierig, ausreichend Arbeitskräfte für die Landwirtschaft zu finden. Immer weniger Menschen sind bereit die körperliche Arbeit und lange Arbeitstage in zumeist dünn besiedelten Regionen Deutschlands auszuführen. Roboter bieten hier die Möglichkeit, diese fehlende Arbeitskraft zu ersetzen. Die mögliche körperliche Entlastung der Landwirt*innen durch Roboter bedingt, dass die Branche geschlechterübergreifend an Attraktivität gewinnen könnte.

Information treatment 3: Environment

Agricultural robots offer great potential to enhance the sustainability and environmental friendliness of agricultural crop production. In the future, the use of robots will allow more targeted management of fields or even of individual plants on a field. This will result in lower quantities of pesticides and fertilizers used. In the future, robots could also be used to grow more than one type of plant in an area, increasing the diversity of fields. Robots are also usually smaller than the tractors and machines commonly used today. This means that small fields can be cultivated, and damage to the soil, such as compaction, can be avoided. In addition, with the relatively smaller robots, it is easier to use drive systems that do not emit carbon dioxide, such as hydrogen or electric drives.

German: Landwirtschaftliche Roboter besitzen großes Potenzial, die landwirtschaftliche Pflanzenproduktion nachhaltiger und umweltfreundlicher zu gestalten. Robotereinsatz erlaubt zukünftig eine gezieltere Bewirtschaftung von Äckern oder sogar von einzelnen Pflanzen auf einem Acker. Dies führt dazu, dass insgesamt geringere Mengen an Pflanzenschutzmitteln und Düngemitteln eingesetzt

werden müssen. Zukünftig könnte durch den Robotereinsatz auch mehr als eine Pflanzenart auf einer Fläche angebaut werden, so dass die Vielfalt auf den Feldern größer wird. Roboter sind zudem meist kleiner als die heute üblichen Traktoren und Maschinen. Dies bedeutet, dass man auch kleine Felder bewirtschaften kann und Schäden am Boden, wie zum Beispiel Verdichtungen, vermieden werden. Zusätzlich ist es bei den im Verhältnis kleineren Robotern einfacher, Antriebssysteme zu verwenden, die kein Kohlendioxid ausstoßen, wie zum Beispiel Wasserstoff- oder Elektroantriebe.

Section 2: Statistics

Supplementary Information Table S1. Knowledge quiz

Statement	Correct in %	Incorrect in %
1 - Wheat is the crop grown in the largest area in Germany. (**True**)	62.36%	37.64%
2-The yields in organic farming systems are substantially lower compared to conventional farming (up to 50 %). (**True**)	73.25%	26.75%
3- Without the application of nitrogen fertilizer, yields in crop farming shrink substantially. (**True**)	70.60%	29.40%
4- In Germany, genetically modified plants are currently cultivated (GM plants). (**False**)	43.06%	56.94%
5- Farmers in Germany are allowed to use as much fertilizer and pesticide as they deem appropriate. (**False**)	92.77%	7.23%
6- The agricultural area in Germany has increased in recent years. (**False**)	76.38%	23.62%
7-Today, tractors can be aligned on the field using GPS on a track given by the computer. They no longer need to be steered. (**True**)	80.21%	19.79%
8- Highly digitized farmers monitor their fields exclusively via satellite photos. They no longer need to check the field personally. (**False**)	73.56%	26.44%

Notes: The table shows the percentage of correct and incorrect responses to the quiz questions.

Supplementary Tables S2a and S2b show the response behavior with regard to selected questions and statements. We present mean values and standard deviations. In addition, we label the statements that have been combined into one factor using PCA for the ABC model and report the internal consistency checks for the other constructs obtained. Furthermore, we report the sampling adequacy and reliability analysis. Re-coded statements due to negative polarization are marked.

Supplementary Information Table S2a. Factor analysis and according descriptive statistics

**Factor MSA, AVE, and reliability**	**Variable**	**Statement**	**Mean**	**SD**	**Factor loadings**[1]
-	Heard of agricultural robots	Have you heard or read anything about the use of robots in agriculture prior to this survey?	1.77	0.66	-
-	Attitude (pre-Frame)	How do you feel about robots being used in agriculture (in the future)?	5.91*	12.15*	-
-	Attitude (post Frame)	Should robots like the ones you have just seen be used in agriculture?	4.11	0.90	-
**ABC (Affective)** KMO: .931***; AVE: 61%, Cα: .911 McDω: 0.918	Scary	I consider the idea of robots autonomously doing field work scary.(-)	3.95	1.14	0.786
**(Affective)**	Benefits	Robots could be of great benefit to agriculture.	4.18	0.86	0.841
**(Affective)**	Ethical	Agricultural robots strike me as questionable from an ethical standpoint(-)	3.94	1.10	0.788
**(Behavioral)**	Purchase intention	I would buy food produced with the help of robots.	4.19	0.95	0.833
**(Behavioral)**	Subsidies	I oppose direct financial support from the state for farmers who use robots in agriculture.(-)	3.68	1.17	0.855
**(Behavioral)**	Surroundings	I would advocate a positive stance towards the use of robots in agriculture in discussions in my surroundings.	3.87	0.99	0.611
**(Cognitive)**	Future	I believe that the use of robot machines is the future of agriculture.	4.07	0.91	0.815
**(Cognitive)**	Research	We should intensify research on agricultural robots and invest in this field.	3.86	0.98	0.830
**(Cognitive)**	System integration	I believe that it is difficult to integrate robots into existing production systems in agriculture.(-)	3.53	1.03	.587

(-)Original statement with negative polarization; Cα and mean refer to ex-post reversion of item
* "Not interested in this topic" responses (n=64) were coded with =77
MSA= Measure of sampling adequacy; KMO= Kaiser, Meyer, and Olkin Test ***=Significant at an .1% α-level; AVE= Average variance extracted; Cα= Cronbach's Alpha; McDω= McDonald's Omega
[1]Extraction method: Principal component analysis (Varimax rotation)

Supplementary Information Table S2b. Component reliability and according descriptive statistics

Factor<br>MSA, AVE, and reliability	Variable	Statements	mean	SD
**Involvement**<br>AVE: 67%,<br>Cα: .87<br>McDω: 0.870	Interest	I have a personal interest in agriculture.	3.28	1.02
	Fascination	Agricultural machinery, such as tractors and combine harvesters, fascinate me.	3.02	1.23
	Conversation	I try to talk to farmers to better understand agriculture.	2.69	1.17
	No interest if safe	I have little interest in learning more about other aspects of production as long as the food is produced safely, i.e., it poses no health risk.(-)	3.43	1.18
	No interest if sustainable	I have little interest in learning more about further aspects of the production as long as the food is produced sustainably.(-)	3.45	1.16
**Trust**<br>AVE: 72%,<br>Cα: .802<br>McDω: 0.808	Environment	How much trust do you have in German farmers regarding environmental protection?	3.16	.91
	Food quality	“”? ….Food quality	3.67	.80
	Animal welfare	“”? ….Animal welfare	2.97	1.01
**Climate-conscious food purchasing behavior**<br>AVE: 64%,<br>Cα: .806<br>McDω: 0.812	Climate-conscious eating	I try to eat in a climate-friendly way.	3.30	1.01
	Climate-friendly production conditions	When I buy food, I hardly pay attention to the stated production conditions (origin, farming methods, cultivation methods, etc.).(-)	3.31	1.20
	Climate impact	From my point of view, it is important to understand the climate impact of our nutrition.	3.73	1.03
	Sustainability	Sustainable food production is, for me, no purchasing argument.(-)	3.33	1.19

(-)Original statement with negative polarization; Cα and mean refer to ex-post reversion of item
MSA= Measure of Sampling Adequacy; AVE= Average variance extracted; Cα= Cronbach’s Alpha; McDω= McDonald’s Omega

Supplementary Information Table S3. Group-specific residency by federal state

Federal state	Control	Food security	Labor	Environment	Sample overall	Germany
Schleswig-Holstein	21 (3.7)	21 (3.7)	21 (3.7)	21 (3.7)	84 (3.7)	3%
Hamburg	14 (2.5)	12 (2.1)	14 (2.5)	14 (2.5)	54 (2.4)	2%
Niedersachsen	56 (9.8)	58 (10.1)	54 (9.6)	55 (9.8)	223 (9.8)	10%
Bremen	5 (0.9)	6 (1.0)	5 (0.9)	5 (0.9)	21 (0.9)	1%
Nordrhein-Westfalen	124 (21.7)	120 (20.9)	125 (22.2)	116 (20.7)	485 (21.4)	22%
Hessen	42 (7.4)	44 (7.7)	42 (7.4)	45 (8.0)	173 (7.6)	7%
Rheinland-Pfalz	27 (4.7)	27 (4.7)	25 (4.4)	29 (5.2)	108 (4.8)	5%
Baden-Württemberg	75 (13.1)	79 (13.8)	74 (13.1)	75 (13.4)	303 (13.3)	13%
Bayern	86 (15.1)	87 (15.2)	83 (14.7)	82 (14.6)	338 (14.9)	15%
Saarland	9 (1.6)	8 (1.4)	8 (1.4)	8 (1.4)	33 (1.5)	1%
Berlin	23 (4.0)	25 (4.4)	24 (4.3)	24 (4.3)	96 (4.2)	4%
Brandenburg	18 (3.2)	17 (3.0)	17 (3.0)	17 (3.0)	69 (3.0)	3%
Mecklenburg-Vorpommern	11 (1.9)	9 (1.6)	12 (2.1)	11 (2.0)	43 (1.9)	2%
Sachsen	28 (4.9)	29 (5.1)	28 (5.0)	27 (4.8)	112 (4.9)	5%
Sachsen-Anhalt	17 (3.0)	15 (2.6)	16 (2.8)	17 (3.0)	65 (2.9)	3%
Thüringen	15 (2.6)	16 (2.8)	16 (2.8)	15 (2.7)	62 (2.7)	3%
χ^2-Test	Pearson χ^2 = 2.5807 Pr = 1.000				-	-
**n**	571	573	564	561	2,269	-
Pearson χ^2 test of independence: Significant differences occur at a Probability (Pr) level <0.05						

Notes: The table presents the residency distribution by federal state across the experimental treatment and control groups. An insignificant chi-square test demonstrates that the groups do not differ significantly with regard to the characteristics, based on a 95% confidence interval.

Supplementary Information Table S4. Dunn-test Post-hoc analysis - Awareness

**Have you ever heard of agricultural robots? – Differences between sociodemographic groups**					
**Age groups**					
Kruskal-Wallis	$\chi2$=5.510 with 4 degrees of freedom; probability=0.2389 (n=2,269)				
**Groups**	**μ (z)**	**r**	**Groups**	**μ (z)**	**r**
18-30 vs. 31-40	1.73/1.76 (-0.8)	-	31-40 vs. 51-60	1.76/1.82 (-1.3)	-
18-30 vs. 41-50	1.73/1.73 (-0.2)	-	31-40 vs. >60	1.76/1.78 (-0.5)	-
18-30 vs. 51-60	1.73/1.82 (-2.2)	-	41-50 vs. 51-60	1.73/1.82 (-2.0)	-
18-30 vs. >60	1.73/1.78 (-1.5)	-	41-50 vs. >60	1.73/1.78 (-1.3)	-
31-40 vs. 41-50	1.76/1.73 (0.7)	-	51-60 vs. >60	1.82/1.78 (0.9)	-
**Income groups**					
Kruskal-Wallis	$\chi2$=23.277 with 3 degrees of freedom; probability=0.0001 (n=2,269)				
**Groups**	**μ**	**r**	**Groups**	**μ**	**r**
<1.500€ vs. 1.501€-3.000€	1.89/1.78*	0.08	1.501€-3.000€ vs. 3.001€-4.500€	1.78/1.76	-
<1.500€ vs. 3.001€-4.500€	1.89/1.76**	0.10	1.501€-3.000€ vs. >4.500€	1.78/1.66**	0.07
<1.500€ vs. >4.500€	1.89/1.66***	0.18	3.001€-4.500€ vs. >4.500€	1.76/1.66*	0.05
**Education**					
Kruskal-Wallis	$\chi2$=16.043 with 2 degrees of freedom; probability=0.0003 (n=2,269)				
**Groups**	**μ**	**r**	**Groups**	**μ**	**r**
Low vs. Middle	1.84/1.76* (2.5)	0.07	Middle vs. High	1.76/1.70 (1.8)	-
Low vs. High	1.84/1.70*** (4.4)	0.11	-	-	-
**Resident (Rural or city)**					
Mann-Withney[1]	z=-2.464; probability=0.0137				
Rural vs. City[1]	1.71/1.79*	0.05	-	-	-

The socio-demographic groups refer to the categories shown in Table 1.
[1]Mann-Whitney-U-Test (Two-samples)
μ= Mean values of the variables for the respective group
z= z-statistic group-wise comparison
r= Wilcoxon Effect Size ($Z/\sqrt{N}$)
*, **, and *** indicate significant differences in mean at the 5%, 1%, and .1% levels

Notes: The Table illustrates the inferential comparison of response behavior between sociodemographic groups. We calculate a Dunn Post-hoc-Test based on the Kruskal-Wallis test.

Supplementary Information Table S5. Attitude models - regression results

	ABC Model (post information)			Attitude (post information)	
	**ABC**	**ABC1**	**ABC2**	**Attitude**	**Attitude1**
	β/SE	β/SE	β/SE	β/SE	β/SE
**Food security[1]**	**1.36** (.16)**	**0.10* (.05)**	**0.12* (.06)**	**1.60*** (.18)**	**0.21*** (.05)**
**Labor[1]**	**1.68*** (.19)**	**0.17*** (.05)**	**0.23*** (.06)**	**1.55*** (.18)**	**0.18** (.05)**
**Environmental[1]**	**1.59*** (.17)**	**0.17*** (.05)**	**0.22*** (.06)**	**1.82*** (.20)**	**0.27*** (.05)**
Trust	1.44*** (.09)	0.15*** (.02)	0.19*** (.03)	1.33*** (.08)	0.13*** (.03)
Involvement	1.22** (.07)	0.08*** (.02)	0.10*** (.03)	1.24*** (.07)	0.09** (.03)
Food purchasing[2]	1.50*** (.10)	0.16*** (.03)	0.19*** (.03)	1.31*** (.09)	0.12*** (.03)
Organic food[3]	0.94 (.05)	-0.02 (.02)	-0.01 (.03)	1.04 (.05)	0.02 (.02)
Male (base= female)	1.50*** (.13)	0.16*** (.03)	0.21*** (.04)	1.41*** (.12)	0.15*** (.04)
Urban residents (b.= rural)	1.34** (.13)	0.13*** (.04)	0.17*** (.05)	1.32** (.13)	0.14** (.04)
East Germans (b.= West)	0.97 (.10)	0.00 (.04)	0.01 (.05)	1.06 (.12)	0.01 (.05)
Low proximity to ag[4]	0.80* (.08)	-0.04 (.04)	-0.06 (.05)	0.83 (.09)	-0.05 (.05)
High proximity to ag[4]	0.72** (.08)	-0.11** (.04)	-0.12* (.05)	0.80 (.09)	-0.07 (.05)
Knowledge (middle)[5]	1.91*** (.37)	0.20* (.08)	0.28** (.10)	1.76** (.33)	0.27** (.09)
Knowledge (high)[5]	2.54*** (.51)	0.31*** (.08)	0.40*** (.10)	2.09*** (.41)	0.37*** (.09)
Stated knowledge	0.92 (.05)	-0.03 (.02)	-0.03 (.03)	0.92 (.05)	-0.04 (.03)
Education (mediocre)[6]	1.14 (.14)	0.08 (.05)	0.11 (.06)	1.11 (.13)	0.09 (.06)
Education (high)[6]	1.57** (.20)	0.16** (.05)	0.23*** (.06)	1.53** (.20)	0.21*** (.06)
Age 31-40[7]	0.93 (.13)	-0.05 (.05)	-0.07 (.07)	0.75* (.11)	-0.11 (.06)
Age 41-50[7]	0.98 (.14)	0.00 (.05)	0.01 (.07)	0.76 (.11)	-0.11 (.06)
Age 51-60[7]	0.89 (.12)	-0.06 (.06)	-0.07 (.07)	0.77 (.11)	-0.14* (.06)
Age >60[7]	1.24 (.17)	0.07 (.05)	0.11 (.07)	0.97 (.14)	-0.02 (.07)
1.500-3.000€[8]	1.28 (.17)	0.09 (.05)	0.11 (.07)	1.12 (.15)	0.08 (.06)
3.001-4.500€[8]	1.78*** (.25)	0.21*** (.06)	0.26*** (.07)	1.60** (.23)	0.22*** (.07)
>4.500€[8]	2.15*** (.33)	0.29*** (.06)	0.36*** (.08)	2.09*** (.32)	0.33*** (.07)
N	2251	2251	2251	2251	2251
Log-Likelihood/R-squared	-2582.95	0.1313	0.1386	-2583.15	0.1210

"ABC"= Ologit using the transformed factor score (rounded) – coefficients displayed as Odds-Ratios
"ABC1"= OLS using the average factor score
"ABC2"= OLS using the Anderson-Rubin factor score
"Attitude"= Ologit using the direct attitude question – coefficients displayed as Odds-Ratios
"Attitude1"= OLS using the direct attitude question
*, **, and *** indicate significance at the 5%, 1%, and 0.1% levels, respectively
[1]Base category=Control group
[2]Climate-conscious food purchasing behavior according to the scale of Brunso et al. 2021
[3]Frequency of organic food purchasing (Likert-type scale 1=never to 5=always)
[4]Base category= No contact (Low= Contact via farm shops and farm holidays High= Contact via job, family or friends)
[5]Base category=Low knowledge (Based on the quiz results: 0-2 Points=low, 3-5=middle, 6-8=high)
[6]Base category= No school-leaving qualification/secondary school
[7]Base category= 18-30
[8]Base category= <1.500€

Supplementary Information Table S6. Distribution of attitude responses by treatment and control groups

Group affiliation					
**ABC**					
Average response to ABC model questions (Fig. 2)	Control	Food Security	Labor	Environment	Total
Strongly disagree (1)	12 (2.10)	8 (1.40)	4 (0.71)	6 (1.07)	30 (1.32)
Rather disagree(2)	28 (4.90)	25 (4.36)	25 (4.43)	12 (2.14)	90 (3.97)
Partly/Partly (3)	136 (23.82)	123 (21.47)	95 (16.84)	108 (19.25)	462 (20.36)
Rather agree (4)	278 (48.69)	250 (43.63)	263 (46.63)	276 (49.20)	1067 (47.03)
Strongly agree (5)	117 (20.49)	167 (29.14)	177 (31.38)	159 (28.34)	620 (27.32)
Total	571	573	564	561	2,269
**Attitude**					
Should robots like the ones you have just seen be used in agriculture?	Control	Food Security	Labor	Environment	Total
Strongly disagree (1)	14 (2.45)	7 (1.22)	7 (1.24)	5 (0.89)	33 (1.45)
Rather disagree (2)	22 (3.85)	18 (3.14)	22 (3.90)	15 (2.67)	77 (3.39)
Partly/Partly (3)	122 (21.37)	94 (16.40)	90 (15.96)	73 (13.01)	379 (16.70)
Rather agree (4)	247 (43.26)	217 (37.87)	212 (37.59)	231 (41.18)	907 (39.97)
Strongly agree (5)	166 (29.07)	237 (41.36)	233 (41.31)	237 (42.25)	873 (38.48)
Total	571	573	564	561	2,269
Notes: Number of responses in each group and response category shown with percentage shares in parentheses.					

Supplementary Information Table S7. Marginal effects ordered logistic regressions (control variables)

Variable	Marginal effect Category 1[1]	Marginal effect Category 2[1]	Marginal effect Category 3[1]	Marginal effect Category 4[1]	Marginal effect Category 5[1]
	**ABC/Attitude**	**ABC/Attitude**	**ABC/Attitude**	**ABC/Attitude**	**ABC/Attitude**
Trust	-0.003**/-0.004***	-0.013***/-0.009***	-0.046***/-0.032***	-/-0.016***	0.066***/0.061***
Involvement	-0.005***/-0.003**	-0.007***/-0.007***	-0.025***/-0.024***	-/-0.012***	0.036***/0.046***
Food purchasing	-0.005***/-0.004***	-0.015***/-0.008***	-0.051***/-0.030***	-/-0.015***	0.073***/0.057***
Organic food	-/-	-/-	-/-	-/-	-/-
Male	-0.005***/-0.005**	-0.015***/-0.011***	-0.050***/-0.038***	-/-0.019***	0.072***/0.073***
Urban residents	-0.004**/-0.004*	-0.011**/-0.009**	-0.037**/-0.031**	-/-0.015**	0.053**/0.059**
East Germans	-/-	-/-	-/-	-/-	-/-
Low proximity to ag	0.003*/-	0.008*/-	0.027*/-	-/-	-0.041*/-
High proximity to ag	0.004**/-	0.012**/-	0.040**/-	-/-	-0.059**/-
Knowledge (middle)	-0.012*/-0.011*	-0.031**/-0.022*	-0.086***/-0.069**	-/-0.009*	0.098***/0.112***
Knowledge (high)	-0.015**/-0.013**	-0.040***/-0.027**	-0.120***/-0.088***	-/-0.021**	0.151***/0.150***
Stated knowledge	-/-	-/-	-/-	-/-	-/-
Education (mediocre)	-/-	-/-	-/-	-/-	-/-
Education (high)	-0.006**/-0.006**	-0.016**/-0.013**	-0.056***/-0.048**	-/-0.026**	0.081***/0.093***
Age 31-40	-/-	-/-	-/0.032*	-/-	-/-0.062*
Age 41-50	-/-	-/-	-/-	-/-	-/-
Age 51-60	-/-	-/-	-/-	-/-	-/-
Age >60	-/-	-/-	-/-	-/-	-/-
1.500-3.000€	-/-	-/-	-/-	-/-	-/-
3.001-4.500€	-0.008**/-0.007**	-0.023***/-0.015**	-0.075***/-0.056**	-/-0.022***	0.099***/0.101***
>4.500€	-0.010***/-0.010***	-0.028***/-0.022***	-0.097**/-0.083***	-/-0.046***	0.136***/0.160***

All variables and categories including the base categories according to Table 1 and Supplementary Table S3.

[1]Results based on ordered logistic regression models with attitude as a dependent variable measured in terms of a five-point Likert scale for the attitude model or as a rounded average response for the variables used in the ABC model, ranging from 1: “Strongly disagree” to 5: “Strongly agree”. *, **, and *** indicate significance at the 5%, 1%, and 0.1% levels, respectively.

Supplementary Information Table S8. Dunn-test Post-hoc analysis – Attitude & Issues (Treatment groups)

**Evaluating the information effect per group on "Attitude" (pre and post frame) and the ABC model**					
**Attitude[1] (pre-Frame)**					
Kruskal-Wallis	$\chi 2$=2.395 with 3 degrees of freedom; probability=0.4945 (n=2,205[2])				
**Treatment groups**	**µ (z)**	**r**	**Groups**	**µ (z)**	**r**
C vs. FS	3.86/3.83 (0.2)	-	FS vs. L	3.83/3.90 (-1.2)	-
C vs. L	3.86/3.90 (-0.9)	-	FS vs. Env	3.83/3.80 (0.4)	-
C vs. Env	3.86/3.80 (0.7)	-	L vs. Env	3.90/3.80 (1.6)	-
**Attitude[3] (post-Frame)**					
Kruskal-Wallis	$\chi 2$=29.973 with 3 degrees of freedom; probability=0.0001 (n=2,269)				
**Treatment groups**	**µ (z)**	**r**	**Groups**	**µ (z)**	**r**
C vs. FS	3.93/4.15*** (-4.4)	0.13	FS vs. L	4.15/4.14 (0.1)	-
C vs. L	3.93/4.14*** (-4.3)	0.13	FS vs. Env	4.15/4.14 (-1.0)	-
C vs. Env	3.93/4.21*** (-5.4)	0.16	L vs. Env	4.14/4.21 (-1.1)	-
**ABC**					
Kruskal-Wallis	$\chi 2$=21.757 with 3 degrees of freedom; probability=0.0001 (n=2,269)				
**Groups**	**µ (z)**	**r**	**Groups**	**µ (z)**	**r**
C vs. FS	3.80/3.92* (-2.7)	0.08	FS vs. L	3.92/4.00 (-1.7)	-
C vs. L	3.80/4.00*** (-4.3)	0.13	FS vs. Env	3.92/3.99 (-1.1)	-
C vs. Env	3.80/3.99*** (-3.8)	0.11	L vs. Env	4.00/3.99 (0.6)	-
**Group-wise comparison of respondents' perceptions of ag robot-related issues by their level of concern – according to Figure 6**					
**Unnatural$^{(-)}$**					
Kruskal-Wallis	$\chi 2$= 5.055 with 3 degrees of freedom; probability=0.1678 (n=2,269)				
**Groups**	**µ (z)**	**r**	**Groups**	**µ (z)**	**r**
C vs. FS	2.02/2.03 (-0.2)	-	FS vs. L	2.03/1.91 (2.2)	-
C vs. L	2.02/1.91 (2.0)	-	FS vs. Env	2.03/1.99 (0.7)	-
C vs. Env	2.02/1.99 (0.5)	-	L vs. Env	1.91/1.99 (-1.4)	-
**Scary$^{(-)}$**					
Kruskal-Wallis	$\chi 2$=11.786 with 3 degrees of freedom; probability=0.0082 (n=2,269)				
**Groups**	**µ (z)**	**r**	**Groups**	**µ (z)**	**r**
C vs. FS	3.83/3.90 (-1.1)	-	FS vs. L	3.90/4.03 (-2.0)	-
C vs. L	3.83/4.03** (-3.1)	0.09	FS vs. Env	3.90/4.05 (-1.8)	-
C vs. Env	3.83/4.05* (-2.9)	0.09	L vs. Env	4.03/4.05 (0.1)	-
**Unethical $^{(-)}$**					
Kruskal-Wallis	$\chi 2$=12.100 with 3 degrees of freedom; probability=0.0070 (n=2,269)				
**Groups**	**µ (z)**	**r**	**Groups**	**µ (z)**	**r**
C vs. FS	3.83/3.88 (-0.8)	-	FS vs. L	3.88/4.02 (-2.1)	-
C vs. L	3.83/4.02** (-3.0)	0.09	FS vs. Env	3.88/4.02 (-2.1)	-
C vs. Env	3.83/4.02* (-2.9)	0.09	L vs. Env	4.02/4.02 (0.0)	-
**Data protection$^{(-)}$**					
Kruskal-Wallis	$\chi 2$=1.324 with 3 degrees of freedom; probability=0.7235 (n=2,269)				
**Groups**	**µ (z)**	**r**	**Groups**	**µ (z)**	**r**
C vs. FS	2.67/2.69 (-0.1)	-	FS vs. L	2.69/2.69 (0.8)	-
C vs. L	2.67/2.69 (0.7)	-	FS vs. Env	2.69/2.71 (-0.3)	-
C vs. Env	2.67/2.71 (-0.4)	-	L vs. Env	2.69/2.71 (-1.1)	-
**Rural depopulation$^{(-)}$**					
Kruskal-Wallis	$\chi 2$=5.448 with 3 degrees of freedom; probability=0.1418 (n=2,269)				
**Groups**	**µ (z)**	**r**	**Groups**	**µ (z)**	**r**
C vs. FS	2.73/2.73 (-0.2)	-	FS vs. L	2.73/2.63 (1.7)	-
C vs. L	2.73/2.63 (1.5)	-	FS vs. Env	2.73/2.78 (-0.7)	-
C vs. Env	2.73/2.78 (-0.9)	-	L vs. Env	2.63/2.78 (-2.4)	-
**Job cuts**					
Kruskal-Wallis	$\chi 2$=22.876 with 3 degrees of freedom; probability=0.0001 (n=2,269)				
**Groups**	**µ (z)**	**r**	**Groups**	**µ (z)**	**r**
C vs. FS	3.06/3.10 (-0.6)	-	FS vs. L	3.10/2.80*** (4.5)	0.13
C vs. L	3.06/2.80** (3.9)	0.12	FS vs. Env	3.10/2.96 (2.0)	-
C vs. Env	3.06/2.96 (1.4)	-	L vs. Env	2.80/2.96* (-2.5)	0.07
**Cyber security$^{(-)}$**					
Kruskal-Wallis	$\chi 2$=2.959 with 3 degrees of freedom; probability=0.3981 (n=2,269)				
**Groups**	**µ (z)**	**r**	**Groups**	**µ (z)**	**r**
C vs. FS	3.14/3.12 (0.4)	-	FS vs. L	3.12/3.04 (1.2)	-
C vs. L	3.14/3.04 (1.6)	-	FS vs. Env	3.12/3.12 (-0.3)	-
C vs. Env	3.14/3.12 (0.2)	-	L vs. Env	3.04/3.12 (-1.4)	-

**Higher food prices**					
Kruskal-Wallis	χ2=3.293 with 3 degrees of freedom; probability=0.3487 (n=2,269)				
**Groups**	**μ (z)**	**r**	**Groups**	**μ (z)**	**r**
C vs. FS	3.51/3.47 (0.4)	-	FS vs. L	3.47/3.41 (1.2)	-
C vs. L	3.51/3.41 (1.6)	-	FS vs. Env	3.47/3.43 (1.0)	-
C vs. Env	3.51/3.43 (1.4)	-	L vs. Env	3.41/3.43 (-0.2)	-
**Human component**					
Kruskal-Wallis	χ2=6.496 with 3 degrees of freedom; probability=0.0898 (n=2,269)				
**Groups**	**μ (z)**	**r**	**Groups**	**μ (z)**	**r**
C vs. FS	3.55/3.45 (1.5)	-	FS vs. L	3.45/3.46 (0.0)	-
C vs. L	3.55/3.46 (1.5)	-	FS vs. Env	3.45/3.39 (1.2)	-
C vs. Env	3.55/3.39 (2.6)	-	L vs. Env	3.76/3.39 (1.1)	-

(-)Original statement with negative polarization; mean refers to ex-post reversion of item
[1]Based on the question: How do you feel about robots being used in agriculture (in the future)?
[2]The 64 participants who expressed no interest in the topic were left out of this analysis for comparability of the questions.
[3]Based on the question: Should robots like the ones you have just seen be used in agriculture?
C= Control group; FS= Food security treatment; L= Labor treatment; Env= Environmental Frame
μ= Mean values of the variables for the respective group
z= z-statistic group-wise comparison
r= Wilcoxon Effect Size ($Z/\sqrt{N}$)
*, **, and *** indicate significant differences in mean at the 5%, 1%, and .1% levels

Notes: The Table illustrates the inferential comparison of response behavior between the different experimental treatment and control groups. We calculate a Dunn Post-hoc-Test based on the Kruskal-Wallis test.
